# Death of Dr. T. L. Raines

**Published:** 1884-09

**Authors:** 


### DR. THOMAS L. RAINES.

A few evenings since the Atlanta Medical and Surgical Union, of which the late Dr. Thomas L. Raines was an officer, passed the following tribute of respect:

IN MEMORIAM.

In the unexpected and untimely death of our brother we have lost a most valued member and fellow worker; one whose genial disposition made him the most acceptable of companions, and whose large and kindly heart called forth the warmest friendships in those who knew him best. In character we feel that he was one of the manliest of men; always actuated by a keen sense of honor, he was strictly reliable and conscientious in his relations with those who came under his medical care, and we as his brethren spontaneously and gladly bear witness to the fact that he was thoroughly unselfish and gentlemanly in all his professional dealings. His career, so suddenly and so sadly closed, gave earnest of a very bright and honorable future, and feeling deeply pained at this unwelcome break in our ranks, we take this means, so inadequate and yet sincere, of expressing our appreciation of our late companion, our unfeigned sorrow at his death, and of extending to his now twice stricken mother, to whom he was more than a son, as well as his sorrowing brothers and sisters, our kindliest sympathy and our tenderest regard.

J. C. AVARY, M. D.,<br>

J. D. WILSON, M. D., } Committee.<br>

R. O. COTTER, M. D.

---

Surgeon Joseph J. Woodward, U. S. A., died near Philadelphia on the 17th of August. His valuable labors in connection with the medical history of the war, and his many contributions to medical literature, have given him wide-spread reputation.

---

Prof. E. Jaeger Von Jaxthall, of Vienna, the celebrated oculist is dead. His test types are used by almost every ophthalmologist in the world.

### ITEMS.

Prof. V. H. Taliaferro will make the opening address at the approaching session of the Atlanta Medical College.

Dr. George H. Noble, a prominent young gynecologist of this city, made his first ovariotomy a few days ago, removing a tumor weighing twenty pounds. At this writing, twenty days after the operation, the patient is up and doing well.

A surgical infirmary has been opened in this city by Dr. J. McF. Gaston, recently removed here from Brazil. Dr. Gaston is a thorough gentleman, learned in the profession, and will no doubt meet with the success which he deserves.

We learn with regret that Dr. John Thad. Johnson, one of the most prominent surgeons of Atlanta, has resigned the professorship of Surgery in the Southern Medical College of this city, and that he will probably remove to California.

The July issue of the *American Journal of the Medical Sciences* contains a handsome picture of the late Prof. S. D. Gross.

The *Pacific Medical and Surgical Journal* and the *Western Lancet*, of San Francisco, have consolidated, with Drs. Henry Gibbons and W. S. Whitwell editors and proprietors. This is the only journal on the Pacific coast devoted to the interests of the regular profession, and deserves to be liberally patronized.

We had a pleasant call a few days ago from Dr. G. W. Holmes, of Rome. He was called to the city to visit a sick relative.

The Bellevue Hospital Medical College, New York, will hereafter issue its diplomas in English instead of Latin.

Dr. J. McF. Gaston, of this city, is making some interesting experiments on dogs, looking to establishing a communication between the gall-bladder and the upper intestines in cases of obstruction to the bile duct. He has an interesting article in this issue of THE JOURNAL, giving results of some of these experiments.

Dr. Koch has been decorated with the cross of the Legion of Honor by the French Government.

